# Preparation of Robust Hydrogen Evolution Reaction Electrocatalyst WC/C by Molten Salt

**DOI:** 10.3390/nano10091621

**Published:** 2020-08-19

**Authors:** Pengpeng Yan, Yuchen Wu, Xiaofeng Wei, Xuewei Zhu, Wei Su

**Affiliations:** 1 College of Mechanical and Electronic Engineering, Northwest A&F University, Yangling 712100, China; yanpengpeng@nwafu.edu.cn (P.Y.); wyc20152019@nwafu.edu.cn (Y.W.); zxw_83614@163.com (X.Z.); 2 School of Physic and Electronic Science, Zunyi Normal University, Zunyi 563000, China

**Keywords:** tungsten carbide, HER, molten salt, stability, application

## Abstract

Tungsten carbide (WC) is an alternative to the costly and resource-constrained Pt-based catalysts for hydrogen evolution reaction (HER). In this work, a one-step facile and easily scalable approach is reported, to synthesize ultrafine WC by molten salt. Benefiting from the ideal synergistic catalytic effect between the highly active WC nanoparticles and the conductive graphitic carbon, and strong charge transfer ability, the unique WC/C hybrids demonstrated excellent HER performance in both acid and alkaline medias with overpotentials of 112 and 122 mV, at a current density of 10 mA cm^−2^ and Tafel slopes of 54.4 and 68.8 mV dec^−1^, in acid and alkaline media, and remarkable stability. With the simplicity and low-cost of the synthetic approach, the strategy presented here can be extendable to the preparation of other transition metal-based/carbon hybrids for versatile applications.

## 1. Introduction

Hydrogen is a good alternative for reducing the energy crisis and protecting the environment [1,2,3]. By using renewable energy to generate electricity, we can produce hydrogen and oxygen by electrolysis of water with catalyst [3,4,5]. Platinum-group metals (PGMs) such as Pt, Rh, and Pd are the most excellent catalysts toward hydrogen evolution reaction (HER) [6,7]. However, the scarcity and high cost of Pt-based electrocatalysts seriously hinder its widespread applications [8,9].

Transition-metal carbides (TMCs) are a good alternative to Pt in HER applications [10,11]. Tungsten carbide (WC) shows excellent activity and stability in acid media because its electronic structures resemble Pt’s [12,13]. However, the HER catalytic activity of WC is inferior to that of Pt-group metals, owing to its higher hydrogen-binding energy (HBE) [14]. Thus, further improvements in the electrocatalytic activity of WC catalyst are urgent and imperative. Nanostructure and adding carbon products are good ways [15,16,17]. Heteroatom dopants, such as N, S, and P, for electronic configuration regulation, are also an efficient method to optimize the physicochemical property of active sites [18,19,20], resulting in improving the HER catalytic activity of WC.

However, utilizing conventional powder-metallurgy route to obtain nano-WC is difficult because sintering and agglomeration of WC inevitably occur at high temperatures (1400–1600 °C) [21,22]. Several approaches to synthesize nano-WC have been developed, including in situ solid reaction [23], combustion carbothermal reduction [24], metal–organic polymeric precursor route [25], and the hard-template method [26]. In general, these methods have the following drawbacks: lengthy preparation process, high temperature, and high energy consumption. Indeed, developing a facile and inexpensive method to obtain nano-WC electrocatalyst remains to be a challenge.

The molten salt synthesis (MSS) is a well-established and low-cost-technique approach which has been developed and used to prepare metal carbides and carbon materials in a flux of low melting point. MSS has the advantage of preventing grain aggregation, reducing reaction temperature, shortening reaction time, and saving energy. Yang et al. [27] synthesized WC + W_2_C powder with 300–500 nm crystal size by NaCl/KCl salt melt at 1000 °C. Glucose was converted into nanoporous carbon and graphitic carbon in LiCl/KCl molten salt, at 300–900 °C [28,29].

Herein, we developed a one-step pyrolysis method to synthesize ultrafine nitrogen–sulfur (N–S)-doped WC catalyst supported on carbon sheet surface via a NaCl/KCl molten salt synthesis method. In this work, ammonium metatungstate (AMT) ((NH_4_)_6_H_2_W_12_O_40_ *x*H_2_O), glucose (C_6_H_12_O_6_), dicyanodiamide (C_2_H_4_N_4_), and cysteine (C_3_H_7_NO_2_S) were used as W, C, N, and S sources, respectively [30,31,32]. It is worth mentioning that this developed method, without any hard template or hydrocarbon gas feeding, is, notably, very facile and efficient with low cost. In addition, the as-obtained N–S-doped WC/C manifests excellent electrocatalytic activity and durability for HER in acid and alkaline media. The as-synthesized products show a highly dispersed distribution of nanoparticles on the carbon sheet surface, which provides a large surface area and high conductivity, thus increasing the HER catalytic activity and stability.

## 2. Materials and Methods

### 2.1. Chemicals and Reagents

Ammonium metatungstate (AMT) was bought from the Grand Sea Tungsten & Molybdenum Group Co. Ltd. (Ganzhou, China). Glucose, dicyandiamide, cysteine, NaCl, and KCl were purchased from Sinopharm Chemical Reagent Co. Ltd. (Shanghai, China). Nafion D-521 dispersion (5 wt %) and Pt/C (20 wt %) were obtained from Alfa Aesar (Shanghai, China). All the solutions in this work were prepared with deionized water.

### 2.2. Material Synthesis

AMT (1 g), glucose (1.25 g), dicyandiamide (1.875 g), and cysteine (0.2 g) were dissolved into 12 mL of deionized water. After stirring for 10 min, 25 g of NaCl/KCl (molar ratio of NaCl/KCl = 1:1) was added into the above solution and stirred for 30 min. Then, the obtained mixture was completely dried in a 60 °C oven, for 10 h, and the resultant solid mixture was ground into very fine powder. After pyrolysis at 900 °C for 1 h, with a ramp rate of 5 °C min^−1^, in N_2_ atmosphere, the obtained product was repeatedly washed with deionized water, to remove the NaCl/KCl under sonication and centrifugalization. Finally, the black powders were harvested and dried at 50 °C, for 4 h, and the WC/C hybrids were readily obtained. Samples without dicyandiamide and cysteine (denoted as WC/C) were also synthesized by the above procedure. For the synthesis of the WC/C electrocatalyst, the mass ratio of AMT (1 g) and glucose (1.25 g) was defined as 4:5; thus, the electrocatalyst was denoted as 4 AMT (WC/C). Similarly, 0.75 and 1.25 g AMT were also used with 3:5 and 5:5 initial mass ratios of AMT and glucose, to prepare the samples by a similar procedure, and the obtained samples were denoted as 3 and 5 AMT (WC/C), respectively. The purchased Pt/C (20 wt %) was used for comparison catalysts. Supplementary Materials Figure S1 demonstrates a typical synthesis process of the WC nanoparticles. To reveal the underlying chemical reaction with AMT, glucose, dicyandiamide, and cysteine, the related equations can be summarized in Supplementary Materials Equation (S1).

### 2.3. Characterization

X-ray diffraction (XRD) was operated by D8 ADVANCE A25 (Bruker, Karlsruhe, Germany). Scanning electron microscopy (SEM) was implemted on Nano SEM-450 (FEI, Hillsborough, OR, USA). Transmission electron microscopy (TEM) and energy-dispersive X-ray spectroscopy (EDXS) mapping were carried out on a TALOS F200X (FEI, Hillsborough, OR, USA). X-ray photoelectron spectroscopy (XPS) was measured on an AXIS UltraDLD (Shimadzu, Kyoto, Japan). An ASAP 2020 analyzer was used to obtained the nitrogen adsorption–desorption isotherms (Micromeritics, Norcross, GA, USA).

### 2.4. Electrochemical Measurements

Electrochemical evaluations were operated in 0.5 M H_2_SO_4_ and 1 M KOH solution with CHI660E instrument (Chenhua, Shanghai, China) by a standard three-electrode. KCl saturated calomel electrode (SCE) and carbon rod were used as the reference electrode and the counter electrode, respectively. Then, 5 mg of catalyst was mixed with 0.5 mL of deionized water, 0.5 mL of ethanol, and 20 µL of 5 wt % Nafion in a 1.5 mL vial, then conducted by ultrasonication for 60 min to form well-dispersed catalyst ink. Then, 10 µL catalyst suspensions were coated on the clean surface of the glassy carbon electrode (GCE) with the diameter of 3 mm. Then, the electrode was dried under an infrared lamp, in air, before measurement, and the loading amount of WC/C was calculated to be 0.71 mg cm^−2^.

Linear sweep voltammetry (LSV) was performed (between 0.15 and −0.5 V vs. RHE) at scan rate of 5 mV s^−1^. All potentials were referenced to a reversible hydrogen electrode (RHE): E (RHE) = E (SCE) (saturated calomel electrode) +0.241 V +0.059 pH.

Electrochemical impedance spectroscopy (EIS) measurements were performed from 100 kHz to 0.1 Hz, with an amplitude of 10 mV. The double-layer capacitance (C_dl_) of catalysts was conducted in the region (0.1–0.3 V vs. RHE). The cyclic voltammetry (CV) curves for C_dl_ were evaluated at different scan rates (25–300 mV s^−1^). All the LSV curves data manifested were amended with IR compensations. All potential values in paper are relative to the RHE. The schematic illustrations regarding the HER activities of the catalysts in acidic and alkaline media, are provided, respectively, in Supplementary Materials Figure S2.

## 3. Results and Discussion

**Figure 1:**
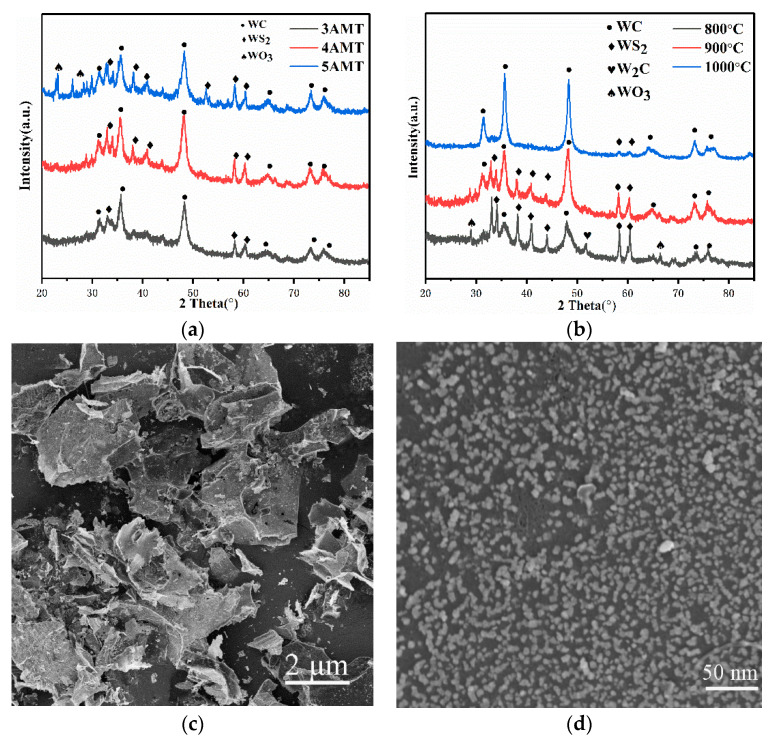
(**a**) X-ray diffraction (XRD) images of 4 ammonium metatungstate (AMT) prepared at different AMT–glucose ratios at 900 °C. (**b**) XRD images of 4 AMT at different temperatures and (**c**,**d**) scanning electron microscopy (SEM) images of 4 AMT.

Figure 1a shows the XRD patterns of as-synthesized WC/C samples with different AMT–glucose ratios at 900 °C. As can be seen, WC is more probably to be formed when the ratio of AMT/C_6_H_12_O_6_ is lower, whereas three smaller peaks of WS_2_ appear in the sample of 3 AMT. Notably, no other characterization peaks, such as W and W_2_C, are observed in the XRD image of 4 AMT. The XRD characteristic peaks of 4 AMT at 31.4°, 35.5°, 48.2°, 65.6°, 73.0°, and 77.0° correspond to the (001), (100), (101), (002), (111), and (102) planes, which is consistent with the standard cards of WC (JCPDS no. 51-0939). There are several peaks originated form WS_2_. A small amount of WS_2_ has synergistic effect with WC [32,33]. At a relatively high AMT/C_6_H_12_O_6_ ratio (5 AMT), two smaller peaks of WO_3_ are observed, indicating that WC, WS_2_, and WO_3_ can coexist at this temperature. Figure 1b compares the XRD images of 4 AMT catalyst pyrolyzed at different synthesis temperatures (800–1000 °C). At 900 and 1000 °C, the W_2_C and WO_3_ phase disappear and the diffraction peaks along the WC phase are enhanced. With increased temperature, carbon atoms continue to diffuse into the sample lattice, and WS_2_ is further converted into WC. At a relatively low temperature (800 °C), a smaller peak of W_2_C is observed, indicating that W_2_C, WO_3,_ WS_2_, and WC can coexist at this temperature.

**Figure 2:**
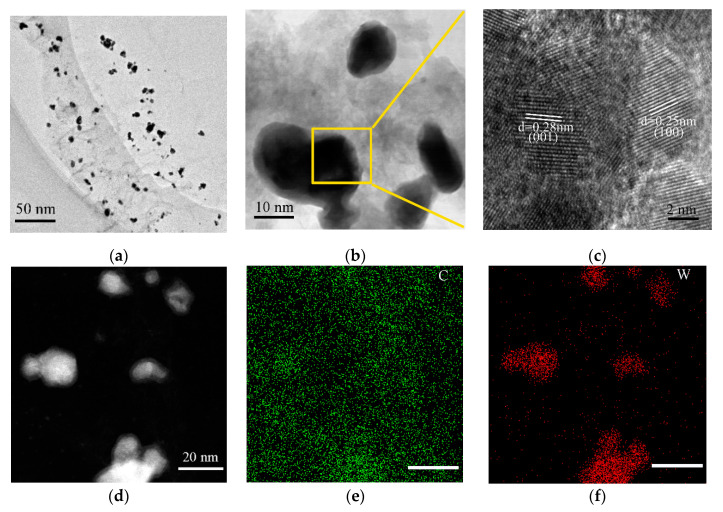
(**a**,**b**) Different magnification TEM images, (**c**) high-resolution TEM image, (**d**) high-angle annular dark-field (HAADF) image, and (**e**,**f**) EDXS mapping images of W and C in 4 AMT.

At different magnifications, the morphology of 4 AMT was first characterized by SEM, as shown in Figure 2c,d. WC presents uniform, sphere-like, and monodisperse small-scale nanoparticles. The average diameter size and its distribution values of the nanoparticles are shown in Supplementary Materials Figure S3. As you can see, the well-dispersed WC nanoparticles are embedded on flake-like carbon sheets surface, which helps to further improve the catalytic active sites of HER exposure quantity. This structure prevents the WC nanoparticles from aggregating and migrating in the process of catalytic reaction and helps to improve the electrical conductivity of catalyst. Different AMT ratios (Supplementary Materials Figure S4) have an important effect on the final morphology of the products. It can be explained that 3 AMT does not contain as much WC nanoparticles as 4 AMT. Nevertheless, excessive W content in the precursor could result in WC coarsening and a loss of surface porosity. As shown in Supplementary Materials Figure S5, the content of W increases with the addition of AMT, while the content of N and S does not change much. As shown in Supplementary Materials Figure S6, the sintering temperatures have an important effect on the final morphology of the 4 AMT. No obvious WC nanoparticles were formed at 800 °C. The WC nanoparticles are growth, particle coarsening, and agglomeration at 1000 °C.

**Figure 3:**
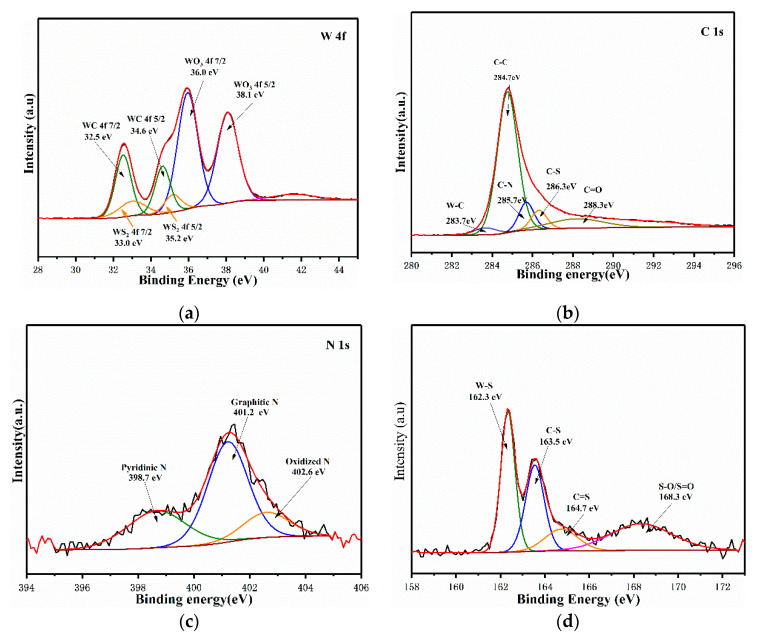
High-resolution XPS spectra of (**a**) W 4f, (**b**) C 1s, (**c**) N 1s, and (**d**) S 2p.

To further reveal the structure and morphology of 4 AMT, it was characterized by TEM. Results are shown in Figure 2. Figure 2a,b shows that WC manifests a spherical particle structure, which is consistent with the SEM results. Thus, the surface of the carbon sheet is loaded with nanoparticles with small particle average diameter. The particle average diameter of WC is about 15 nm. Flake carbon provides excellent electrical conductivity and prevents active ingredients from leaching in a corrosive environment [34]. Figure 3c shows clear crystal lattice fringes with lattice spacing of 0.247 and 0.286 nm, which are corresponding to the (001) and (100) crystal planes of WC. To reveal the element distribution of the 4 AMT, HAADF and EDXS mapping technologies were employed. As can be seen in Figure 2d–f, C and W are distributed in WC nanoparticles. This result also suggests that WC is distributed on the carbon sheet.

XPS was implemented to further gain more insights into the valence state and structure information of 4 AMT. As shown in Supplementary Materials Figure S7, the full XPS spectra manifest the existence of C, W, O, N, and S elements in the catalyst. In the high-resolution XPS energy spectra of W 4f, there are two pairs of peaks. The first pair of peaks at lower binding energy (32.5 eV for W 4f 7/2 and 34.6 eV for W 4f 5/2) can be ascribed to WC. The second pair of peaks at higher binding energy (36.0 eV for W 4f 7/2 and 38.1 eV for W 4f 5/2) can be ascribed to WO_3_ due to the unavoidable oxidized of WC nanoparticles when exposed to air. The high-resolution C 1s spectra can be fitted with five peaks centered at 283.7, 284.7, 285.7, 286.3, and 288.3 eV, which can be ascribed to C–W, C–C, C–N, C–S, and C=O species, respectively. The high-resolution N 1s spectra can be fitted with four peaks centered at pyridinic-N (398.7 eV), graphitic-N (401.2 eV), and oxidized-N (402.6 eV).The high-resolution S 2p spectra can be fitted with four contributions from the corresponding peaks centered at W–S (162.3 eV), C–S (163.5 eV), C=S (164.7 eV), and oxidized–S (168.3 eV). Compared to C, N or S is more electronegative, which makes the adjacent carbon atoms possessing a higher positive charge density. Accordingly, these adjacent carbon atoms turn into new active sites [35,36,37]. In addition, due to the dopants of N–S on carbon matrix, inner electronic structure of the WC may be regulated by increasing electron density in the carbon matrix, thereby weakening the binding strength of WC-H_ads_ and promoting the H_ads_ desorption during the process of HER [38]. Among the N1s and S2p spectra, the peaks of graphitic-N and graphitic-S are observed, indicating that N and S atoms are successfully doped into the WC nanoparticles. As shown in Figure S8, the full XPS spectra manifest the presence of C, W, and O elements in the WC/C, suggesting that there are no N and S atoms in the WC nanoparticles.

N_2_ adsorption–desorption isotherms were implemented to determine the specific surface area of as-prepared products. As shown in Supplementary Materials Figure S9, specific surface areas of 4 AMT and no N–S-doped WC/C catalysts are 337.3 and 157.6 m^2^ g^−1^. Notably, the high specific surface area can provide more active surface sites and boost HER activity. Without N–S doped, the WC/C catalyst manifests lower specific surface area, which further highlights the important role of N–S in the synthesis of WC/C catalyst. In the process of molten salt synthesis, the formation of molten salt enhances the fluidity of reaction components and significantly increases the diffusion rate. Liquid molten salt penetrates the synthetic powder particles, which can effectively prevent the agglomeration of particles. In addition, the coarsening and growth of WC nanoparticles are inhibited by lower reaction temperature. Therefore, the specific surface area of WC/C is increased. Pore distribution generates from the large amount of gas releasing during the carbon-thermal reaction.

**Figure 4:**
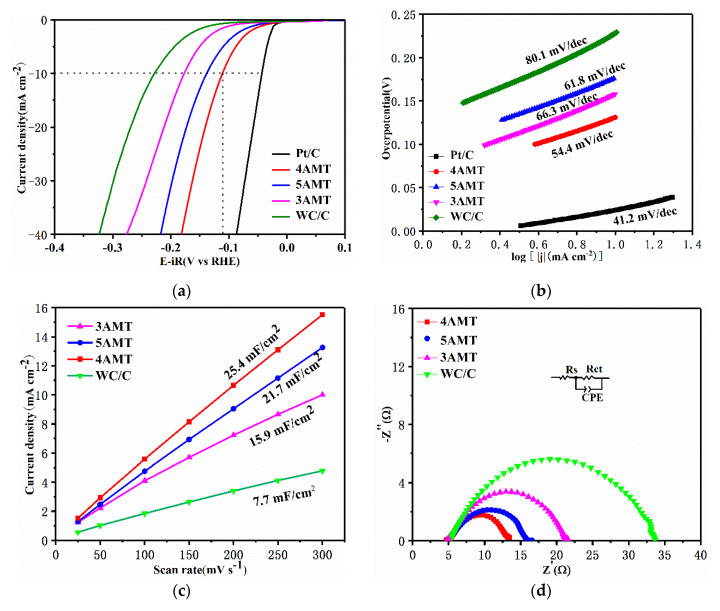

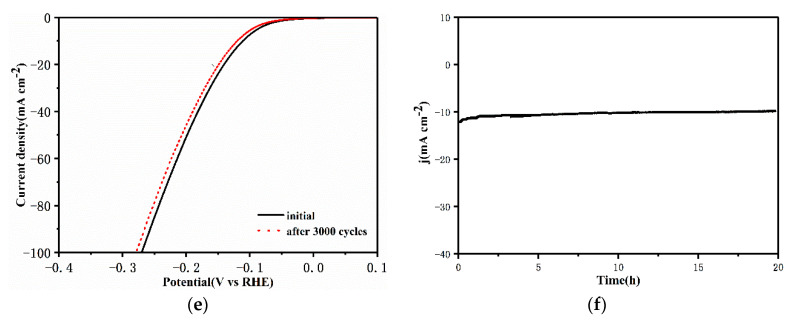
(**a**) LSV curves of 4 AMT, 5 AMT, 3 AMT, WC/C, and Pt/C in 0.5 M H_2_SO_4_; (**b**) Tafel slopes generated from (**a**,**c**) C_dl_ of 4 AMT, 5 AMT, 3 AMT, and WC/C; (**d**) EIS curves of 4 AMT 5 AMT, 3 AMT, and WC/C (insertion is the equivalent circuit); (**e**) LSV curve of 4 AMT after 3000 CV cycles; (**f**) i-t curve of 4 AMT with an overpotential of 120 mV.

Electrochemical measurements were performed to verify the HER activities of the hybrid catalysts synthesized at different AMT ratios in 0.5 M H_2_SO_4_. Figure 4a demonstrates LSV curves of electrocatalyst, along with the property of 20% Pt/C catalyst for reference. The 20% Pt/C catalyst exhibits superior HER activity with an overpotential of 43 mV at 10 mA cm^−2^. Among the prepared catalysts, the 4 AMT catalyst manifests optimal HER property with an overpotential of 112 mV at 10 mA cm^−2^, lower than that of 5 AMT (139 mV), 3 AMT (177 mV), and WC/C (229 mV). The HER catalytic activity of 4 AMT is superior to WCs in Supplementary Materials Table S1.

To discern the HER mechanism of the as-prepared electrocatalysts, Tafel plots were fitted to the Tafel equation (*η* = *a* + *b*log|*j*|, where *j* is the current density and b is the Tafel slope), as shown in Figure 4b. As a rule, Tafel, Heyrovsky, and Volmer step is corresponded to the Tafel slopes of 30, 40, and 116 mV dec^−1^, respectively. The Tafel slope of 4 AMT catalyst was calculated to be 54.4 mV/dec, lower than that of 5 AMT (61.8 mV/dec), 3 AMT (66.3 mV/dec), and WC/C (80.1 mV/dec). The Tafel slope values of the as-prepared electrocatalysts suggest the HER rate is co-determined by the discharge of H_2_O molecule (Volmer mechanism) and the desorption of bonded hydrogen atoms (Heyrovsky mechanism) from the catalyst surface [39,40].

The HER activity of catalyst can be evaluated directly through its active surface area. In general, the active surface area could be represented by the C_dl_ of the catalyst. Figure 4c demonstrates the CV curves of prepared catalysts at different scan rates (25–300 mV s^−1^). The C_dl_ is calculated from the plot of *Δj* = (*j*_a_ − *j*_c_)/2 (where *j*_a_ and *j*_c_ are current densities at 0.24 V vs. SCE) against the scan rate, as shown in Supplementary Materials Figure S10. The C_dl_ of 4 AMT, 5 AMT, 3 AMT, and WC/C is calculated to be 25.4, 21.7, 15.9, and 7.7 mF cm^−2^, respectively. In addition, the electrochemically active surface area (ECSA) can be calculated by using the following equation: ECSA = (C_dl_/m)/60 μF cm^−2^, where m is the areal loading amount of the catalyst (0.71 mg cm^−2^). As a result, the ECSA of 3 AMT, 4 AMT, 5 AMT, and WC/C is calculated to be 37.3, 59.6, 50.9, and 18.1 m^2^ g^−1^, respectively, indicating that there are more active sites in the 4 AMT electrocatalyst. Obviously, the WC/C catalyst possesses the lowest ESCA, which should be attributed to the coupled effects of small specific surface area and no N–S mass loading.

EIS was also carried out to manifest the electrocatalytic kinetics. As shown in Figure 4d, an equivalent circuit model with three parts are used to analyze the HER process. Rs and CPE represent the solution resistance and capacitance, respectively. Rct is on behalf of charge-transfer resistance. The Rct of 4 AMT (8.7 Ω) is smaller than that of 5 AMT (11.6 Ω), 3 AMT (16.5 Ω), and WC/C (28.2 Ω), suggesting the faster charge-transfer kinetics process and excellent HER activity of 4 AMT.

Apart from electrocatalytic activity, long-term stability is another important electrochemical performance of 4 AMT. As shown in Figure 4e, the overpotential of 4 AMT at 10 mA cm^−2^ shows a slight decline of 8 mV after 3000 CV cycles, demonstrating its robust stability in acid electrolyte. As shown in Figure 4f, the current density almost remains stable for 20 h. This result manifests that 4 AMT possesses long-term stability in acid media, which is probably attributed to highly stable WC, the special nanostructure, and the protective impact of carbon sheets.

**Figure 5:**
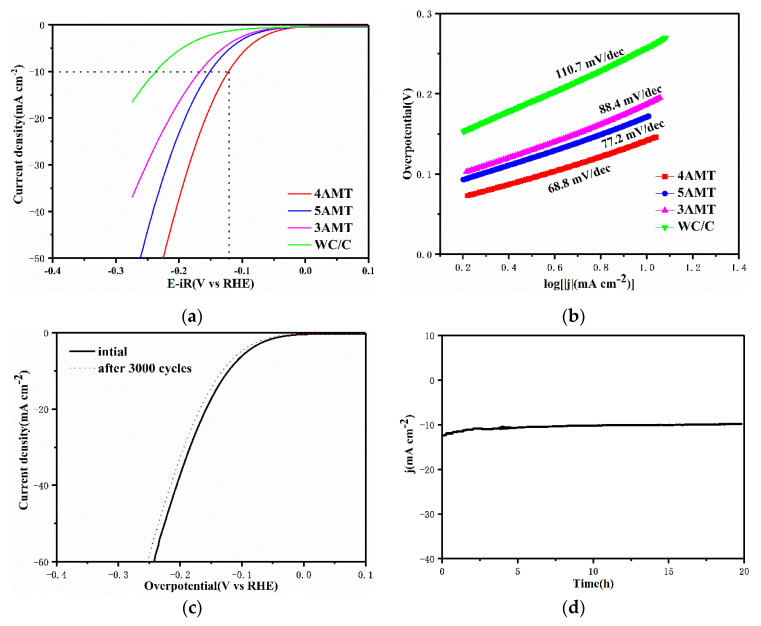
(**a**) LSV curves of 4 AMT, 5 AMT, 3 AMT, and WC/C in 1 M KOH; (**b**) Tafel slopes generated from (**a**,**c**) LSV curve of 4 AMT, before and after 3000 CV cycles; (**d**) i-t curve of 4 AMT with an overpotential of 130 mV.

Because HER is often operated in alkaline electrolyte for actual applications, HER activities for catalysts synthesized at different AMT ratios are also evaluated in 1 M KOH media. Figure 5a shows the LSV curves of 3 AMT, 4 AMT, 5 AMT, and WC/C. As shown in Figure 5a, the 4 AMT catalyst shows excellent HER property with overpotential of 122 mV at 10 mA cm^−2^, which is smaller than that of WC/C (237 mV). Furthermore, Figure 5b demonstrates the Tafel slopes of the 4 AMT (68.8 mV dec^−1^) and WC/C (110.7 mV dec^−1^), which suggests that HER process complies with Volmer–Heyrovsky mechanism. By applying CV cycles and long-term tests at the fixed overpotentials (Figure 5c,d), the stability evaluations were carried out. Results manifest that a high stability for two stability tests is achieved in alkaline electrolyte. The LSV curve of 4 AMT shows no distinct decline after 3000 CV cycles, and the current density almost remains stable for 20 h.

## 4. Conclusions

N–S-doped WC/C electrocatalysts for HER were successfully prepared at 900 °C via a facile one-step synthesis route assisted by KCl + NaCl salt. The addition of KCl + NaCl salt effectively eliminated the runaway expansion during calcination. Apart from molten salt, dicyanodiamide and cysteine play an important role in the prepared WC/C electrocatalysts. They serve as precursors the for N–S-doped carbon matrix and promote the formation of the WC phase. The outstanding electrocatalytic performance for hydrogen production in both alkaline and acidic conditions can be mainly attributed to the optimal synergistic catalytic effect between the highly active WC nanoparticles and the conductive graphitic carbon and fast charge transport ability. Such a facile approach offers a general methodology for design and preparation of transition metal-based/carbon nanosheet hybrids for versatile applications.

## Supplementary Materials

The following are available online at https://www.mdpi.com/2079-4991/10/9/1621/s1. Figure S1: The whole reaction process. Equation S1: The related equations for the further molten salt reactions for the carburization of AMT into WC. Figure S2: The schematic illustration, regarding the HER activities of the catalysts (a) in acidic media and (b) alkaline media. Figure S3: The size and its distribution values of the nanoparticles. Figure S4: SEM images of (a) 3 AMT and (b) 5 AMT. Figure S5: EDX images of XAMT. Figure S6: SEM images of (a) 4 AMT at 800 °C and (b) 4 AMT at 1000 °C. Figure S7: The full XPS spectra of 4 AMT. Figure S8: (a) The full XPS spectra of WC/C (without N–S), (b) W 4f, and (c) C 1s. Figure S9: N_2_ adsorption–desorption isotherm curves of (a) 4 AMT and (b) without N–S-doped WC/C. Figure S10: CV curves of (a) 3 AMT, (b) 4 AMT (c) 5 AMT, and (d) WC/C at different scanning rates, over potential range of 0.1–0.3 V vs. RHE in 0.5 M H_2_SO_4_. Table S1: Comparison of 4 AMT with other WC catalyst in 0.5 M H_2_SO_4_.

## References

[1] Hisatomi T., Kubota J., Domen K. (2014). ChemInform Abstract: Recent advances in semiconductors for photocatalytic and photoelectrochemical water splitting. Chem. Soc. Rev..

[2] Gholipour M.-R., Dinh C.-T., Bél F., Do T.-O. (2015). Nanocomposites as sunlight-driven photocatalysts for hydrogen production from water splitting. Nanoscale.

[3] Feng Q., Yuan X.-Z., Liu G., Wei B., Zhang Z., Li H., Wang H. (2017). A review of proton exchange membrane water electrolysis on degradation mechanisms and mitigation strategies. J. Power Sources.

[4] Wang M., Wang Z., Gong X. (2014). The intensification technologies to water electrolysis for hydrogen production—A Review. Renew. Sustain. Energy Rev..

[5] Wang L., Tranca D.C., Zhang J. (2017). Toward activity origin of electrocatalytic hydrogen evolution reaction on carbon-rich crystalline coordination polymers. Small.

[6] Lei J.-M., Peng Q.-X., Luo S.-P. (2018). A Nickel Complex, An efficient co-catalyst for both electrochemical and photochemical driven hydrogen production from Water. Mol. Catal..

[7] Zhang K., Zhao Y., Fu D. (2015). Molybdenum carbide nanocrystal embedded n-doped carbon nanotubes as electrocatalysts for hydrogen generation. J. Mater. Chem. A.

[8] Lu C., Tranca D., Zhang J., Hernandez F.-R., Su Y., Zhuang X., Zhang F., Seifert G., Feng X. (2017). Molybdenum carbide-embedded nitrogen-doped porous carbon nanosheets as electrocatalysts for water splitting in alkaline media. ACS Nano.

[9] Garcia-Esparza A.-T., Cha D., Ou Y., Kubota J., Domen K., Takanabe K. (2013). Tungsten carbide nanoparticles as efficient cocatalysts for photocatalytic overall water splitting. ChemSusChem.

[10] Liu Q., Shi J., Hu J., Asiri A.-M., Luo Y., Sun X. (2015). CoSe_2_ nanowires array as a 3D electrode for highly efficient electrochemical hydrogen evolution. ACS Appl. Mater. Interface.

[11] Zhang L.-N., Ma Y.-Y., Lang Z.-L., Wang Y.-H., Khan S.-U., Yan G., Tan H.-Q., Zang H.-Y., Li Y.-G. (2018). Ultrafine cablel-like WC/W_2_C heterojunction nanowires covered by graphitic carbon towards highly efficient electrocatalytic hydrogen evolution. J. Mater. Chem. A.

[12] Peppernick S.-J., Gunaratne K.-D., Castleman A.-W. (2010). Superatom spectroscopy and the electronic statec correlation between elements and isoelectronic molecular counterparts. Proc. Natl. Acad. Sci. USA.

[13] Chen W.-F., Muckerman J.-T., Fujita E. (2013). Recent developments in transition metal carbides and nitrides as hydrogen evolution electrocatalysts. Chem. Commun..

[14] Greeley J., Jaramillo T.-F., Bonde J., Chorkendorff I., Nørskov J.-K. (2006). Computational high-throughput screening of electrocatalytic materials for hydrogen evolution. Nat. Mater..

[15] Zheng Y., Jiao Y., Li L.-H., Xing T., Chen Y., Jaroniec M., Qiao S.-Z. (2014). Toward design of synergistically active carbon-based catalysts for electrocatalytic hydrogen evolution. ACS Nano.

[16] Zhao Y., Kamiya K., Hashimoto K., Nakanishi S. (2013). Hydrogen evolution by tungsten carbonitride nanoelectrocatalysts synthesized by the formation of a tungsten acid/polymer hybrid in situ. Angew. Chem. Int. Ed..

[17] Ji L., Wang J., Teng X., Dong H., He X., Chen Z. (2018). N,P-doped molybdenum carbide nanofibers for efficient hydrogen production. ACS Appl. Mater. Interfaces.

[18] Ma Y.-Y., Lang Z.-L., Yan L.-K., Wang Y.-H., Tan H.-Q., Feng K., Xia Y.-J., Zhong J., Liu Y., Kang Z.-H. (2018). Highly efficient hydrogen evolution triggered by a multi-interfacial Ni/WC hybrid electrocatalyst. Energy Environ. Sci..

[19] Feng Q., Xiong Y.-H., Xie L.-J., Zhang Z., Lu X., Wang Y., Yuan X.-Z., Fan J.-T., Li H., Wang H.-J. (2019). Tungsten carbide encapsulated in grape-like N-doped carbon nanospheres: One-step facile synthesis for low-Cost and highly active electrocatalysts in proton exchange membrane water electrolyzers. ACS Appl. Mater. Interfaces.

[20] Lu J., Yin S., Shen P.-K. (2019). Carbon-encapsulated electrocatalysts for the hydrogen evolution reaction. electrochem. Energy Rev..

[21] Wu Z., Yang Y., Gu D., Li Q., Feng D., Chen Z., Tu B., Webley P.-A., Zhao D. (2009). Silica-templated synthesis of ordered mesoporous tungsten carbide/graphitic carbon composites with nanocrystalline alls and high surface areas via a temperature programmed carburization route. Small.

[22] Yan Y., Zhang L., Qi X., Song H., Wang J.-Y., Zhang H., Wang X. (2012). Template-Free pseudomorphic synthesis of tungsten carbide nanorods. Small.

[23] Chen W.-F., Schneider J.-M., Sasaki K., Wang C.-H., Schneider J., Iyer S., Iyer S., Zhu Y., Muckerman J.-T., Fujita E. (2014). Tungsten carbide-nitride on graphene nanoplatelets as a durable hydrogen evolution electrocatalyst. ChemSusChem.

[24] Chen Z., Qin M.-L., Chen P.-Q., Jia B.-R., He Q., Qu X.-H. (2016). Tungsten carbide/carbon composite synthesized by combustion-carbothermal reduction method as Melectrocatalyst for hydrogen evolution reaction. Int. J. Hydrogen Energy.

[25] Xu Y.-T., Xiao X.-F., Ye Z.-M., Zhao S.-L., Shen R.-G., He C.-T., Zhang J.-P., Li Y.-D., Chen X.-M. (2017). Cage-confinement pyrolysis route to ultrasmall tungsten carbide nanoparticles for efficient electrocatalytic hydrogen evolution. J. Am. Chem. Soc..

[26] Hunt S.-T., Nimmanwudipong T., Roman L.-Y. (2014). Engineering non-sintered, metal-terminated tungsten carbide nanoparticles for catalysis. Angew. Chem. Int. Ed..

[27] Yang R.-S., Xing T.-Y., Xu R.-B., Li M.-T. (2011). Molten salt synthesis of tungsten carbide powder using a mechanically activated powder. Int. J. Refract. Met. Hard Mater..

[28] Liu X.-F., Cristina G., Markus A. (2014). A Facile Molten-Salt Route to graphene synthesis. Small.

[29] Li X.-H., Kurasch S., Kaiser U., Antonietti M. (2012). Synthesis of monolayer-patched graphene from Glucose. Angew. Chem. Int. Ed..

[30] Han N.-N., Yang K.-R., Lu Z.-Y., Li Y.-J., Xu W.-W., Gao T.-F., Cai Z., Zhang Y., Baista V.-S., Liu W. (2018). Nitrogen-doped tungsten carbide nanoarray as an efficient bifunctional electrocatalyst for water splitting in acid. Nat. Commun..

[31] Zhang L.-P., Yang H.-B., Wanigarathna D., Li B. (2018). Ultrasmall transition metal carbide nanoparticles encapsulated in N,S-doped graphene for all-pH hydrogen evolution. Small Methods.

[32] Nguyn T.-P., Kim S.-Y., Lee T.-H., Jang H.-W., Lee Q.-V., Kim I.-T. (2020). Facile Synthesis of W_2_C@WS_2_ Alloy Nanoflowers and their Hydrogen Generation Performance. Appl. Surf. Sci..

[33] Li Y., Wu X., Zhang H., Zhang J. (2018). Interface designing over WS_2_/WC for enhanced hydrogen evolution catalysis. ACS Appl. Energy Mater..

[34] Gao Y., Lang Z., Yu F., Tan H., Yan G., Wang Y., Ma Y., Li Y. (2018). A Co_2_P/WC Nano-heterojunction covered with N-doped carbon as highly efficient electrocatalyst for hydrogen evolution reaction. ChemSusChem.

[35] Zou X., Huang X., Goswami A., Silva R., Sathe B.-R., Mikmekova E., Asefa T. (2014). Cobalt-embedded nitrogen-rich carbon nanotubes efficiently catalyze hydrogen evolution reaction at all pH values. Angew. Chem. Int. Ed..

[36] Liu Y., Yu G., Li G.-D., Sun Y., Asefa T., Chen W., Zou X. (2015). Coupling Mo_2_C with nitrogen-rich nanocarbon leads to efficient hydrogen-evolution electrocatalytic sites. Angew. Chem..

[37] Deng J., Ren P., Deng D., Bao X. (2015). Enhanced electron penetration through an ultrathin graphene layer for highly efficient catalysis of the hydrogen evolution reaction. Angew. Chem. Int. Ed..

[38] Wang S., Wang J., Zhu M., Bao X., Xiao B., Su D., Li H., Wang Y. (2015). Molybdenum-carbide-modified nitrogen-doped carbon vesicle encapsulating nickel nanoparticles: A highly efficient, low cost catalyst for hydrogen evolution reaction. J. Am. Chem. Soc..

[39] Hussain S., Akbar K., Vikraman D., Afzal R.A., Song W., An K.S., Farooq A., Park J.Y., Chun S.H., Jung J. (2018). WS_(1-X)_Se_X_ nanoparticles decorated three-dimensional graphene on nickel foam: A robust and highly efficient electrocatalyst for the hydrogen evolution reaction. Nanomaterials.

[40] Sajjad H., Jinwoong C., Kamran A., Dhanasekaran V., Linh T., Bilal A.-N., Yawar A., Hyun S.-K., Seung H.-C., Gunn K. (2019). Fabrication of robust hydrogen evolution reaction electrocatalyst using Ag_2_Se by vacuum evaporation. Nanomaterials.

