# Supplementary material for: Preparation of Robust Hydrogen Evolution Reaction Electrocatalyst WC/C by Molten Salt
Source: Nanomaterials (Basel). 2020 Aug 19;10(9):1621. doi: 10.3390/nano10091621 (PMC7559515; doi:10.3390/nano10091621)
Supplement: Supplementary file 1 [file nanomaterials-10-01621-s001.pdf]

Supplementary Materials

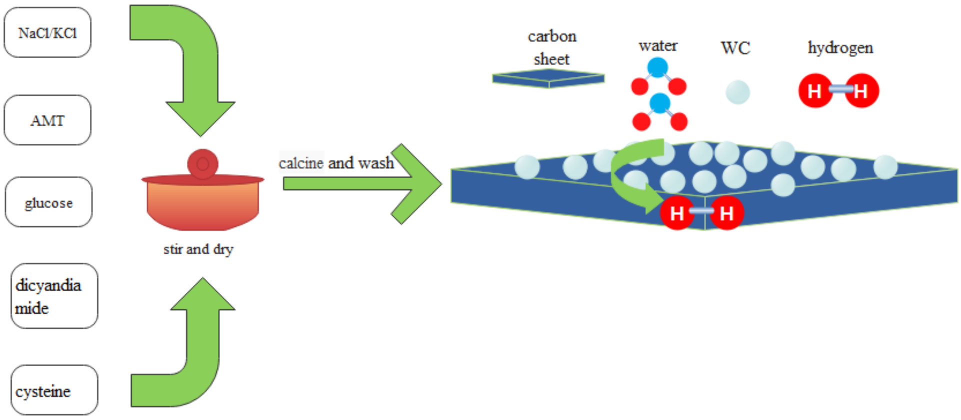

Figure S1. The whole reaction process.

$(NH_4)_6H_2W_{12}O_{40}{\cdot}xH_2O+C_6H_{12}O_6+C_2H_4N_4+C_3H_7NO_2S\rightarrow WO_3+NO+NO_2+CO+CO_2+SO+SO_2+H_2O$ (1)

$WO_3 + CO\rightarrow \quad W + CO_2$ (2)

$CO\rightarrow \quad C+CO_2\ SO\rightarrow \quad S+SO_2$ (3)

$W+(1-X)C\rightarrow \quad WC_{(1-X)} \quad WC_{(1-X)}\rightarrow WC_2+WC \quad WC_2+C\rightarrow \quad WC$ (4)

Equation S1. The related equations for the further molten salt reactions for the carburization of AMT into WC.

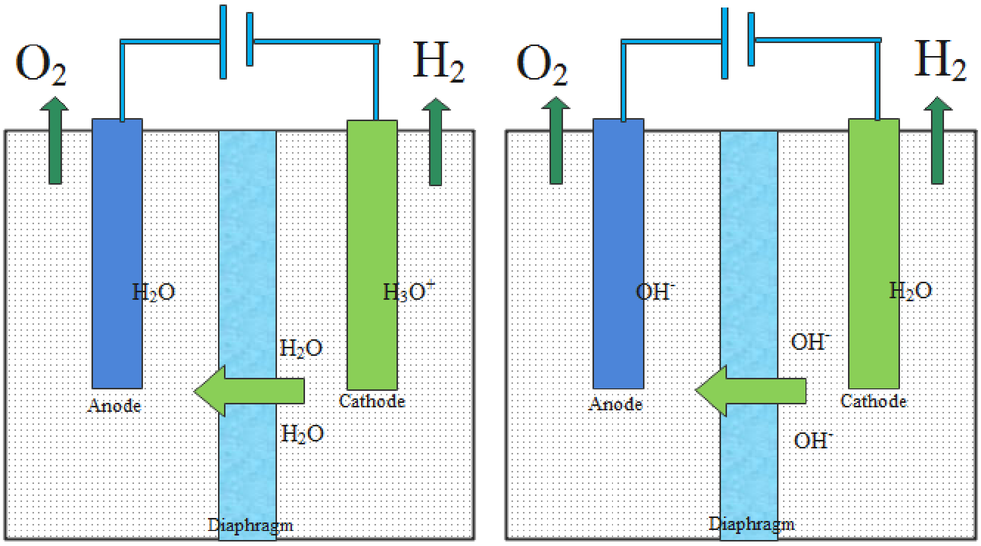

(a) (b)

Anode: $H_2O\rightarrow \quad 1/2O_2+2H^{+}+2e^{-}$

Cathode: $2H_3O^{+}+2e^{-}\rightarrow \quad H_2+2H_2O$

Anode: $2OH^{-}\rightarrow \quad 1/2O_2+H_2O+2e^{-}$

Cathode : $2H_2O+2e^{-}\rightarrow \quad H_2+OH^{-}$

Figure S2. The schematic illustration, regarding the HER activities of the catalysts (**a**) in acidic media and (**b**) alkaline media.

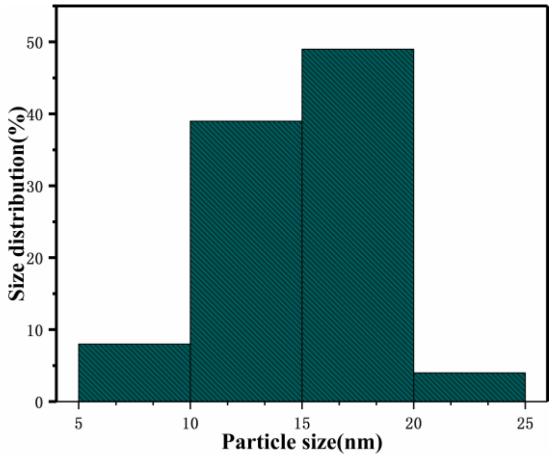

Figure S3. The size and its distribution values of the nanoparticles.

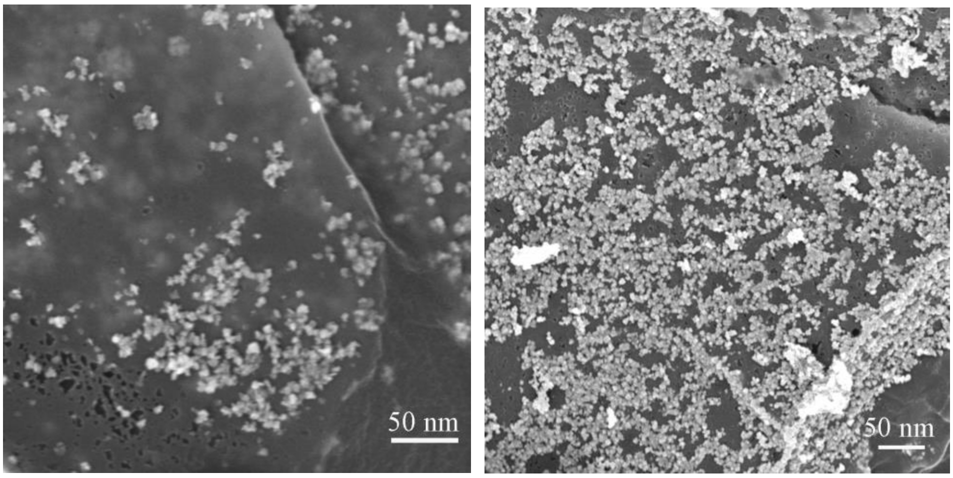

(a) (b)

Figure S4. SEM images of (**a**) 3AMT and (**b**) 5AMT.

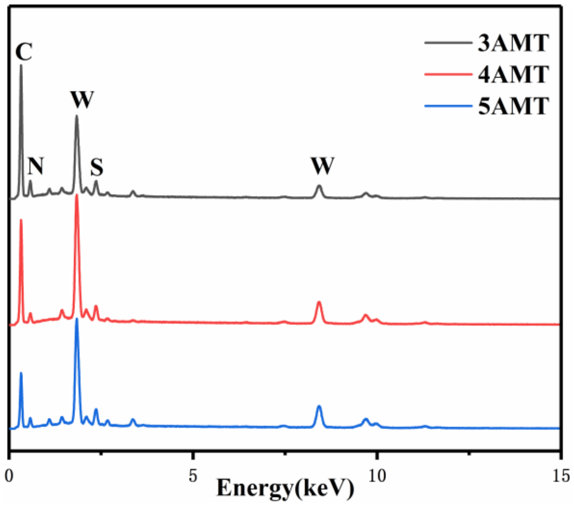

Figure S5. EDX images of XAMT.

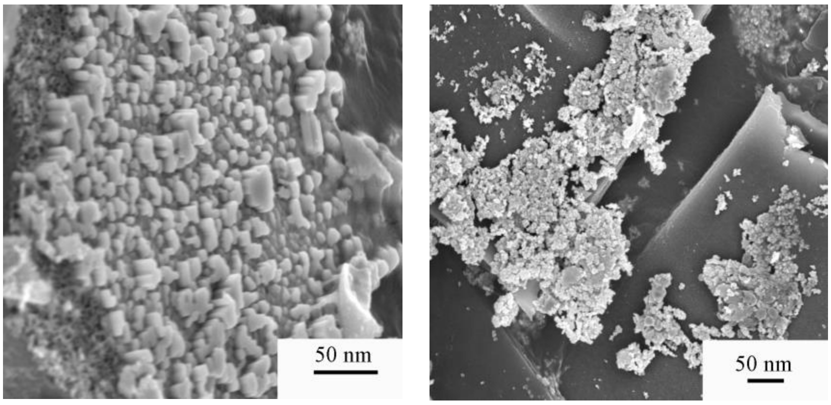

(a) (b)

Figure S6. SEM images of (**a**) 4AMT at 800 °C and (**b**) 4AMT at 1000 °C.

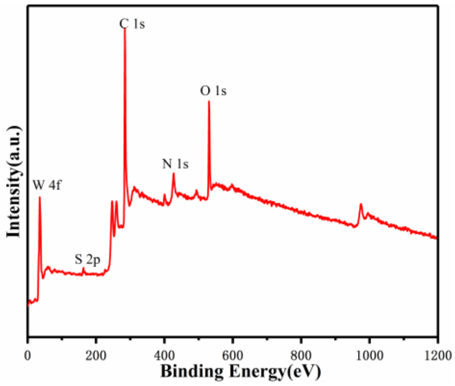

Figure S7. The full XPS spectra of 4AMT.

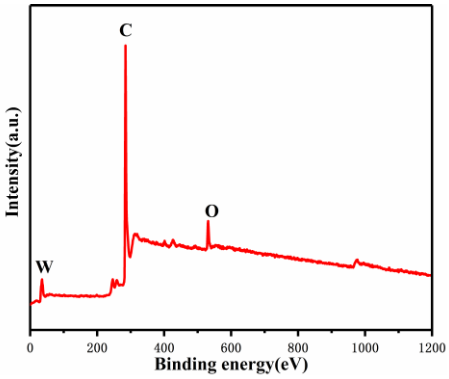

(a)

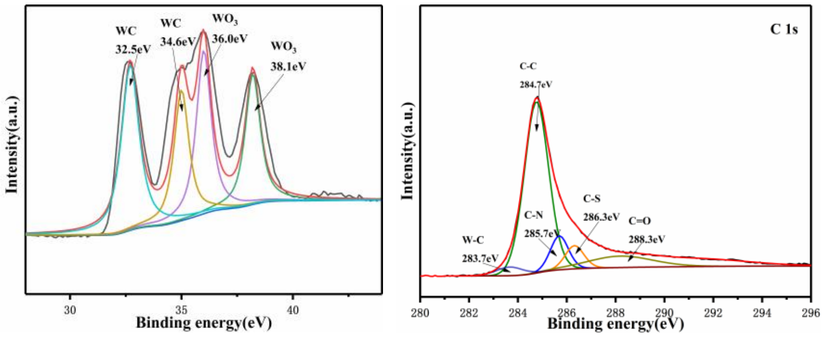

(b) (c)

Figure S8. (**a**) the full XPS spectra of WC/C(without N-S), (**b**) W 4f, (**c**) C 1s.

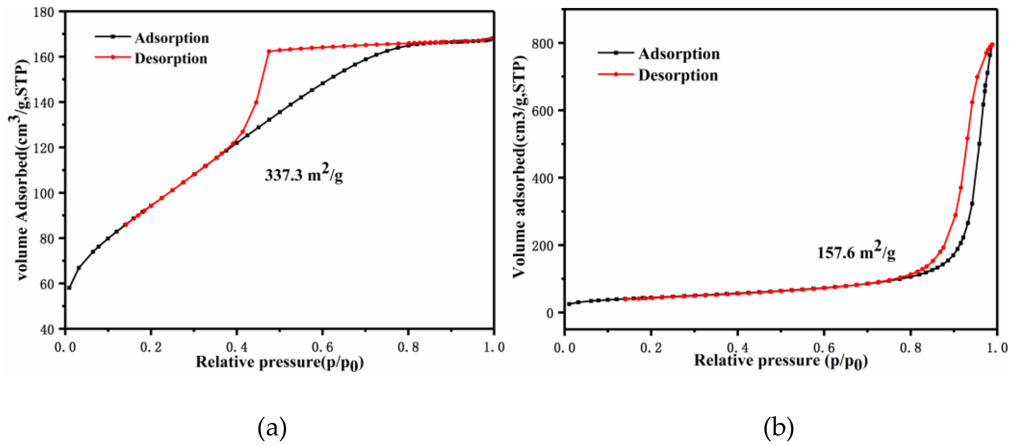

Figure S9. N2 adsorption-desorption isotherm curves of (**a**) 4AMT and (**b**) without N-S-doped WC/C.

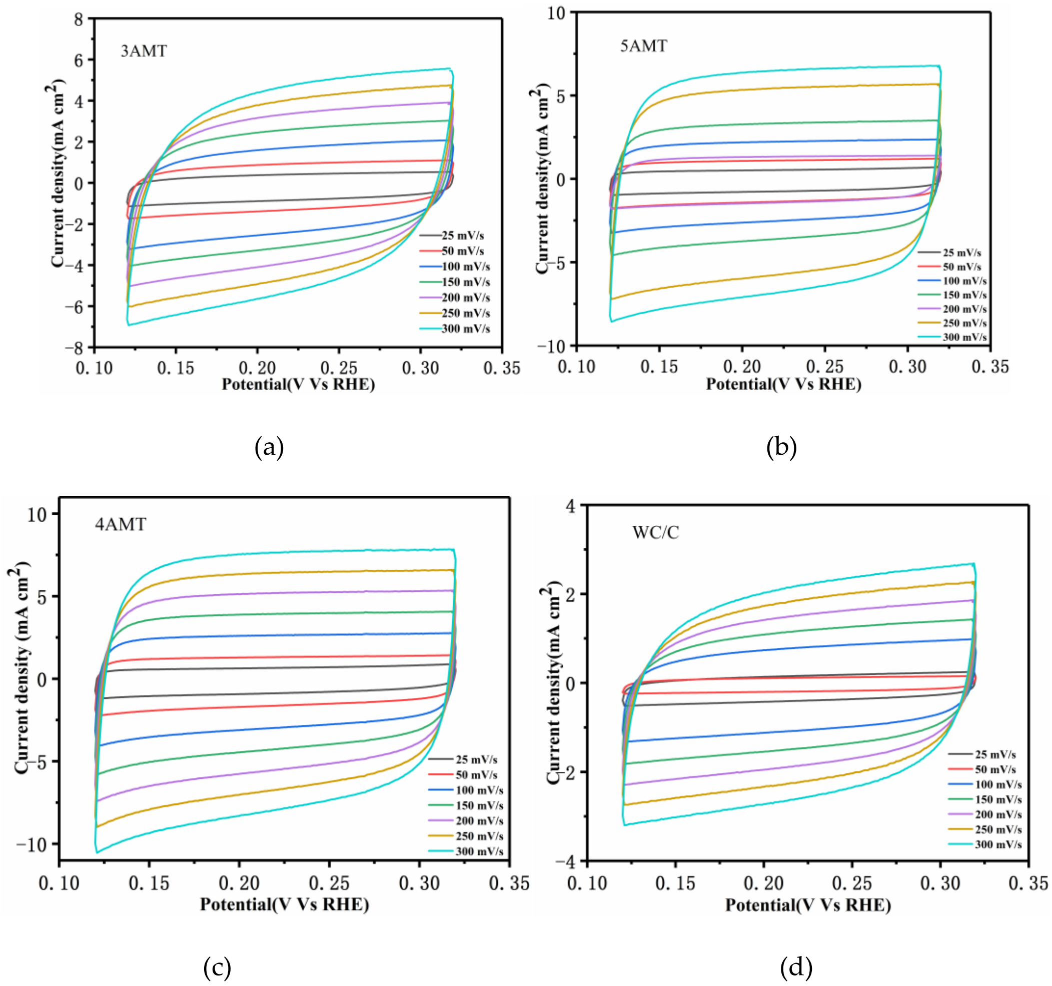

Figure S10. CV curves of (**a**) 3AMT,(**b**) 4AMT (**c**) 5AMT, (**d**) WC /C at different scanning rates over potential range of 0.1-0.3 V vs. RHE in 0.5 M H_2SO_4.

Table S1. Comparison of 4AMT with other WC catalyst in 0.5 M H_2SO_4.

Material	Media	Overpotential	Tafel slope	Reference
WWC@NC	0.5M H_2SO_4	127	56.3	[19]
WC/CNT	0.5M H_2SO_4	250	78	Nat. Commun. 2016, 7, 13216
Porous WC	0.5M H_2SO_4	187.1	87.8	Nanoscale 2017, 9, 5413
H-WC	0.5M H_2SO_4	160	102.2	Electrochimica Acta, 2019, 319, 775
5%Pt/WC/C_{10}	0.5M H_2SO_4	128	42	Appl. Surf. Sci., 2019, 463, 1154
